# MiRNA therapeutics based on logic circuits of biological pathways

**DOI:** 10.1186/s12859-019-2881-7

**Published:** 2019-11-22

**Authors:** Valeria Boscaino, Antonino Fiannaca, Laura La Paglia, Massimo La Rosa, Riccardo Rizzo, Alfonso Urso

**Affiliations:** CNR-ICAR, National Research Council of Italy, via Ugo La Malfa 153, Palermo, 90146 Italy

**Keywords:** miRNA therapeutics, Drug discovery, Cancer pathway, Logic circuit, Boolean network

## Abstract

**Background:**

In silico experiments, with the aid of computer simulation, speed up the process of in vitro or in vivo experiments. Cancer therapy design is often based on signalling pathway. MicroRNAs (miRNA) are small non-coding RNA molecules. In several kinds of diseases, including cancer, hepatitis and cardiovascular diseases, they are often deregulated, acting as oncogenes or tumor suppressors. miRNA therapeutics is based on two main kinds of molecules injection: miRNA mimics, which consists of injection of molecules that mimic the targeted miRNA, and antagomiR, which consists of injection of molecules inhibiting the targeted miRNA. Nowadays, the research is focused on miRNA therapeutics. This paper addresses cancer related signalling pathways to investigate miRNA therapeutics.

**Results:**

In order to prove our approach, we present two different case studies: non-small cell lung cancer and melanoma. KEGG signalling pathways are modelled by a digital circuit. A logic value of 1 is linked to the expression of the corresponding gene. A logic value of 0 is linked to the absence (not expressed) gene. All possible relationships provided by a signalling pathway are modelled by logic gates. Mutations, derived according to the literature, are introduced and modelled as well. The modelling approach and analysis are widely discussed within the paper. MiRNA therapeutics is investigated by the digital circuit analysis. The most effective miRNA and combination of miRNAs, in terms of reduction of pathogenic conditions, are obtained. A discussion of obtained results in comparison with literature data is provided. Results are confirmed by existing data.

**Conclusions:**

The proposed study is based on drug discovery and miRNA therapeutics and uses a digital circuit simulation of a cancer pathway. Using this simulation, the most effective combination of drugs and miRNAs for mutated cancer therapy design are obtained and these results were validated by the literature. The proposed modelling and analysis approach can be applied to each human disease, starting from the corresponding signalling pathway.

## Background

MicroRNA (miRNAs) are non coding RNA molecules, 18-22 nucleotide long, responsible of gene regulation at post transcriptional level. They act interacting through their “seed” region, binding the complementary region (typically the 3’Untranslated Region (UTR)) of their RNA messenger (mRNA) target. MiRNA-mRNA interactions lead to mRNA degradation or translation repression. This mechanism is responsible for the control of target gene expression [1].

Moreover, their role as regulators of different physiological and pathological conditions as cancer has been validated [2]. Indeed, their expression is often deregulated in tumors, and they can have an oncogenic or tumor-suppressive role, depending on their target gene [3]. As an example, miR-222 can have a dual role of oncogene or tumor-suppressor in different cell types according to its target gene. miR-222 is over-expressed in lung cancer and it has PTEN tumor suppressor gene as target [4]; on the other hand, it is down-regulated in acute myelogenous leukemia and it has c-kit oncogene as target [5]. In translational field, recent studies considered miRNAs as therapeutic agents because of their regulative role [6]. On the basis of the dual role of miRNAs as oncogenes or tumor-suppressor, there are two possible miRNA-based drugs, called respectively antagomiR and miRNA-mimics. The former are synthetic compounds that inhibit miRNA action, by acting as their antagonists [7], the latter are molecules that mimic miRNAs behaviour [6]. miRNA-mimics were used as cancer therapeutic for the first time in lung cancer [8, 9]. For example, miR-34 reached the phase I trial for cancer treatment and antimiR targeted miR-122 reached phase II trial for hepatitis [10].

Different strategies have already been applied in miRNA therapeutics [1]. As example, sandwich RNA interference (RNAi) inhibition involved the concurrent use of a multiplex of miRNAs to target a single target gene [11]. This strategy has the advantage to exhibit synergistic anti-tumor efficiency than single therapy alone. Another therapeutic approach is the use of a “cocktail” of different miRNAs in order to interact with many targets at the same time [12]. As previously said, miRNA drugs can be divided into miRNA antagomiRs and miRNA mimics. As an example, pre-clinical studies adopted antisense oligonucleotides targeting miRNAs (AMOs) and Locked Nucleic Acid (LNA) anti-miRs in phase I and II trials [7, 13]. Both are duplexes of RNA analogues/miRNAs able to degrade miRNA molecule and to recycle the antagomiR.

miRNA therapeutics is actually carried out by in vitro or in vivo experiments. These experiments are expensive and time consuming. Therefore, in silico experiments play a key role in the development of therapy for several diseases, because they speed up the process of in vivo or in vitro experiments. In this paper, following the same principles of drug discovery experiments based on computer simulations of logical circuits [14], we present a pathway modelling approach based on digital circuits for miRNA therapeutics studies. The proposed modelling and analysis approach can be applied to each cancer pathway. The proposed work aims at offering to biologists and clinicians a method to analyse drugs and miRNAs effects on a specific disease.

## Related works

Recently, several authors proposed novel approaches to analyse signalling pathways based on logic circuits [14–20].

In [14], authors propose the digital approach on the growth factor signalling pathway. Relationships between genes in the pathway are properly modelled using Boolean logic gates. Gene mutations are modelled as faults in the Boolean network. Two type of faults are identified: stuck-at-1 and stuck-at-0. The former provides the disconnection of the faulted net from the upstream gates and the connection of the faulted net to a logic value of 1, meaning that the gene is always expressed regardless of the other signals in the network. In the latter fault, the faulted net is disconnected from the upstream logic gates and connected to a logic value of 0, meaning that the corresponding gene is not expressed. Stuck-at-1 usually represents mutated oncogenes; stuck-at-0 usually represents mutated tumor suppressors. The output of the network is made up of genes playing a key role in the cell proliferation or apoptosis. The analysis of the network is used to enumerate all possible faults and to analyse effects of drugs for single faults in cancer therapy design. Existing data validate the proposed approach.

In [15], the authors address the signalling pathway of castration-resistant prostate cancer and the digital approach is applied to achieve drug discovery. In the literature, only data on a single drug are available and combination with no more than two drugs are considered to avoid excessive toxicity and side-effects. Notwithstanding this, authors propose a combination of three drugs as the most effective. Furthermore, authors propose the stochastic analysis of node vulnerability to identify better targets for drugs.

In [16], the authors propose a digital approach, based on Karnaugh maps, to build a Boolean network whose state transitions model a given biological pathway. A family of Boolean networks is defined which can generate trajectories consistent with given pathway information.

In [17], the authors apply the digital approach to hypoxia stress response pathway to obtain efficient therapeutic interventions. The authors develop a Boolean model with targeted drugs in a cell that mimics persistent hypoxia. The hypoxic pathway is combined with apoptosis, cell survival and energy production processes. Simulation data are confirmed by the literature.

In [18], the authors define a Boolean network to model the cancer growth factor pathway. Faults are identified according to the stuck-at-1 and stuck-at-0 modelling approach. The drug vector is identified and the drug action on the faulted Boolean network is carried out. Multiple faults are accounted for, with two or three simultaneous faults, and for each faulted network. A drug vector is identified, which can completely rectify the fault producing a fault-free output. The drug vector of each faulted network consists of the minimum number of drugs to limit side-effects. Then, the therapy with the fewest drug and best fault coverage is identified based on the circuit analysis.

In [19], the authors focus on MAPK signal transduction network which is modelled by a Boolean network, including target locations of inhibitory drugs. By using a version of the message passage algorithm, the probable locations of dysregulation in the network is obtained.The model results are compared with more extensive Markov chain Montecarlo methods. The proposed method takes advantages from a much smaller computation time. A method to estimate the probability of a certain fault in the Boolean network is therefore proposed.

In [20], the authors develop a model for cancer tissue heterogeneity based on an ensemble of Boolean networks, based on pathway knowledge. The number of networks is equal to the number of major sub-populations in the cancer tissue. The paper aims at finding out the extent to which each network influences the behaviour of the tissue by observing the behaviour of the outputs.

## Methods

In this paper, we present a novel approach to miRNA therapeutics based on logic circuits using the following methodology.

**Fig. 1:**
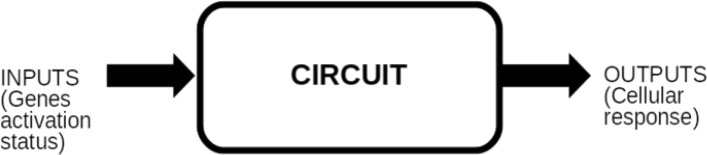
Logic circuit of pathway. In cancer related pathways, inputs are usually growth factors and tumor suppressors. Outputs are usually genes playing a key role in cell proliferation or apoptosis

In a cancer related signalling pathway, the inputs are growth factors and tumor suppressors, the outputs are genes playing a key role in proliferation or apoptosis. From a generic perspective, this type of pathway can be seen as a grey box with input and outputs (Fig. 1). According to the scientific literature presented in the previous section, that grey box will be modelled by a digital circuit. Because these kinds of circuits only account for discrete signals of value zero or one, these circuits can be considered as Boolean networks, and from here on we will use both expressions (digital circuit and Boolean network) indifferently.

**Fig. 2:**
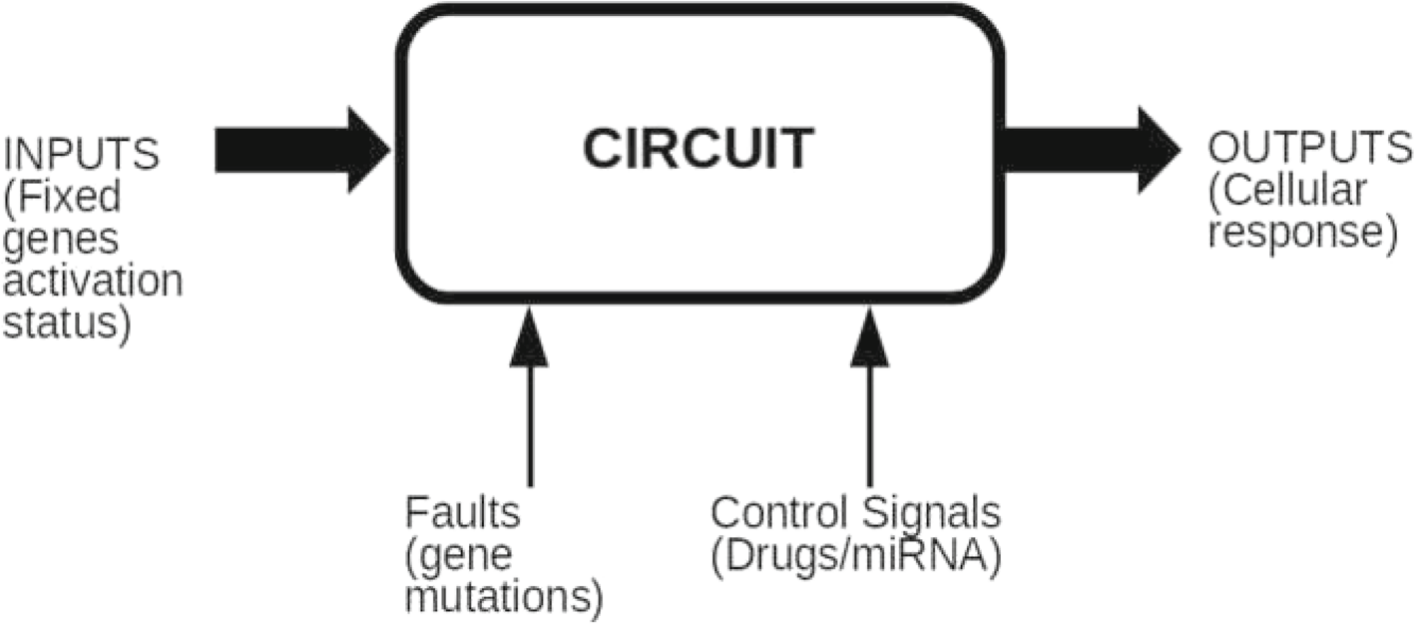
Logic circuit of pathway with mutations and drug/miRNAs. In cancer related pathways, inputs are usually growth factors and tumor suppressors. Outputs are usually genes playing a key role in cell proliferation or apoptosis. Mutations are introduced as perturbations in the digital circuit. Drugs or miRNA are introduced as control signals reducing the circuit outputs to less dangerous condition or even to a fault-free condition

Gene mutations, that alter the pathway responses, are introduced in the circuit as perturbations, meaning that a fault in the Boolean network occurs every time a gene is mutated. These faults are introduced in order to study the effects of mutations on an otherwise healthy organism. After that, the circuit is analysed by fixing the input vector with growth factors set to 0 and tumor suppressors set to 1. This configuration corresponds to the healthy condition of the pathway, i.e. non-proliferative with apoptosis. This way, it is possible to analyse the circuit under each mutation, observing how the mutations alter the output and therefore identifying the most dangerous output condition. In order to change this diseased status, drugs or miRNAs are introduced. They act as control signals, reducing the output to a less dangerous condition or even to a fault-free condition in a complete fault-recovery scenario. Summarizing, the circuit is analysed by fixing the input vector, introducing all the mutations firstly, and those drugs or miRNAs acting on their corresponding target secondly (Fig. 2). The “pathogenic degree” of the output under the action of drugs and miRNAs is computed in order to find the most effective drug or miRNA.

In the following, materials and methods are described in detail. In the “Materials” subsection, we introduce databases and resources used to build and analyse the circuit. In the “Logic circuit of the pathway” subsection, we define the digital circuit and variables, as well as the transformation rules from the biological pathway to the digital circuit. In the “Simulation” subsection, we describe the simulation setup and we define the score used to validate simulation results. The modelling approach and analysis can be applied to each human disease, starting from the corresponding signalling pathway.

### Materials

Biological pathways are retrieved from Kyoto Encyclopedia of Genes and Genomes (KEGG) database [21]. It is a database that integrates genomic, chemical and systemic functional information, converting all the knowledge acquired from different biological data of heterogeneous entities into metabolic pathways.

Mutations are derived from ClinVar [22] and KEGG databases, by introducing all genes involved in the KEGG pathway and selecting the targeted human disease. ClinVar is an NCBI resource, collecting different human clinical variants related to different phenotypes, with supporting evidence. Only pathogenic or likely/pathogenic mutations (mutations with pathological or likely pathological clinical significance) or mutations related to drug response are selected (Table 1).

**Table 1:** NSCLC case study. Gene mutations retrieved from ClinVar. Gene name, accession number, mutation, clinical significance and literature references are reported, respectively

Gene	Accession	Mutation	Clinical significance	Ref
EGFR	NM_005228.4(EGFR)	c.2573T>G (p.Leu858Arg)	Pathogenic-drug response	([61])
ERBB2	NM_001005862.2(ERBB2)	c.2223_2234dupATACGTGATGGC	Pathogenic/Likely pathogenic	([62])
		(p.Ala745_Gly746insTyrValMetAla)		
ALK	NM_004304.4(ALK)	c.3522C>A (p.Phe1174Leu)	Pathogenic/Likely pathogenic	([63])
PTEN	NM_000314.6(PTEN)	c.697C>T (p.Arg233Ter)	Pathogenic	([64])
KRAS	NM_004985.4(KRAS)	c.437C>T (p.Ala146Val)		([65])
BRAF	NM_004333.5(BRAF)	c.1794_1796dup (p.Thr599_Val600insThr)	Pathogenic-drug response	([66])
PI3K	NM_006218.3(PIK3CA)	c.3140A>G (p.His1047Arg)	Pathogenic/Likely pathogenic	([67, 68])
AKT1	NM_005163.2(AKT1)	c.49G>A (p.Glu17Lys)	Pathogenic/Likely pathogenic	([69, 70])
MEK/MAP2K1	NM_002755.3(MAP2K1)	c.167A>C (p.Gln56Pro)	Pathogenic	([71, 72])
EML4-ALK-p16		chr2:29446394..42552694 inversion	Pathogenic	([73])

For each mutated gene, the oncogenic or tumor suppressive role is key to the definition of the digital equivalent circuit. Tumor Suppressor Gene Database (TSGene Database) is a comprehensive database of tumor suppressor genes which is available from the University of Texas, Health Science Center at Houston [23]. Searching the mutated gene in the database is a key preliminary step. Another key step consists of searching the mutated gene in the Network of Cancer Gene (NCG) Database, containing information about the gene function (oncogene or tumor suppressor) of 2372 cancer genes from 273 manually curated publications. NCG is maintained by the Ciccarelli group which is a part of the School of Cancer Studies of King’s College London [24]. Growth factor property for genes of the pathways are retrieved from UniProt db [25].

KEGG provides information about drugs and their targets in the specific pathway under study. We considered those drugs resulting approved or under investigation for that specific cancer on DrugBank database [26]. If present, other drugs provided only by DrugBank are considered.

miRNAs are selected from miRTarBase repository [27]. It allows the exploration of validated miRNA-target interactions, through a collection of different experiment types and literature filtering. Then, the validated miRNA-target couples are filtered for a specific tissue type, shrinking the study to a selected cohort of cases. This last step is achieved through the use of miRTissue service [28]. Moreover it gives information about miRNA behaviour, evidencing both target degradation and protein translation inhibition. The suitable miRNAs are obtained by introducing all pathway genes, selecting “Degradation” as interaction type and filtering results by the selected case study, through p-value thresholds (p-value ≤ 0.05). Moreover, miRNAs post-filtering is required in order to avoid circuit redundancy. To this end, we consider all the miRNAs addressing multiple targets. If a target gene is not covered by the other selected miRNAs, we also consider miRNAs addressing only one target. Finally, we identified clusters of miRNAs addressing the same targets and, in this case, we consider a single representative of the cluster.

### Logic circuit of the pathway

A KEGG pathway is a graph whose nodes are the entries and edges are the relations. The circuit modelling approach is based on the assumptions that nodes can be modelled as Boolean variables and edges by logic gates.

KEGG uses the KEGG Markup Language (KGML) to define entries, properties and relationships in pathways. The KGML Document (which is available at https://www.kegg.jp/kegg/xml/docs/) defines the whole set of the pathway elements. In this paper, we propose a modelling approach for entry and relation types of interest for the specific purpose. The proposed approach aims at modelling the dynamic interactions among genes, enzymes and proteins to make an efficient drug discovery and target discovery analysis for a specific human disease. Since only a subset of the elements included in a KEGG pathway is fundamental for the specific goal, we refer to the KGML Document, extrapolating data of interest and proposing for these elements a suitable modelling approach.

In the KGML Document, entries are classified as: ortholog, enzymes, reactions, genes, groups, compounds, maps, brites and others. Enzymes, genes and compounds are considered as signals in the pathway circuit. Ortholog groups, brites and reactions are not considered for the specific purpose. Orthologs are any of two or more homologous gene sequences found in different species and therefore this information is useless for the specific purpose since a specific human disease is targeted. The BRITE hierarchy file is created by KEGG to represent functional hierarchy of KEGG objects and therefore is useless for the specific goal. Reactions are classified into reversible or irreversible but these information are not useful to model dynamic events and interactions among genes, enzymes and compounds.

In the KGML Document, the relation types are firstly classified as ECrel (enzyme-enzyme), PPrel (protein-protein), GErel (gene expression), PCrel (protein-compound) and maplink (link to another map)but, in this paper, these types of relations are not considered. The circuit modelling is based on subtypes of these relations which lead to a dynamic event. In fact, dynamic events among signals are fundamental for the circuit modelling, no matter which is the biological nature of the specific signal (protein, gene, enzyme, compound). Relations are then classified by subtypes as: compound, hidden compound, activation, inhibition, expression, repression, indirect effect, state change, binding/association, dissociation, missing interaction, phosphorilation, dephosphorilation, glycosylation, ubiquitination and methylation.

The circuit modelling of biological pathways focuses on dynamic events leading to activation, expression, inhibition, repression, binding/association and dissociation. Compounds, hidden compounds and state changes are not accounted for. Compounds are shared with two successive reactions in ECrel or intermediate of two interacting proteins in PPrel. Hidden compounds are shared with two successive reactions but are not displayed in the pathway map. These are steady-state events not related to dynamic actions.

**Fig. 3:**
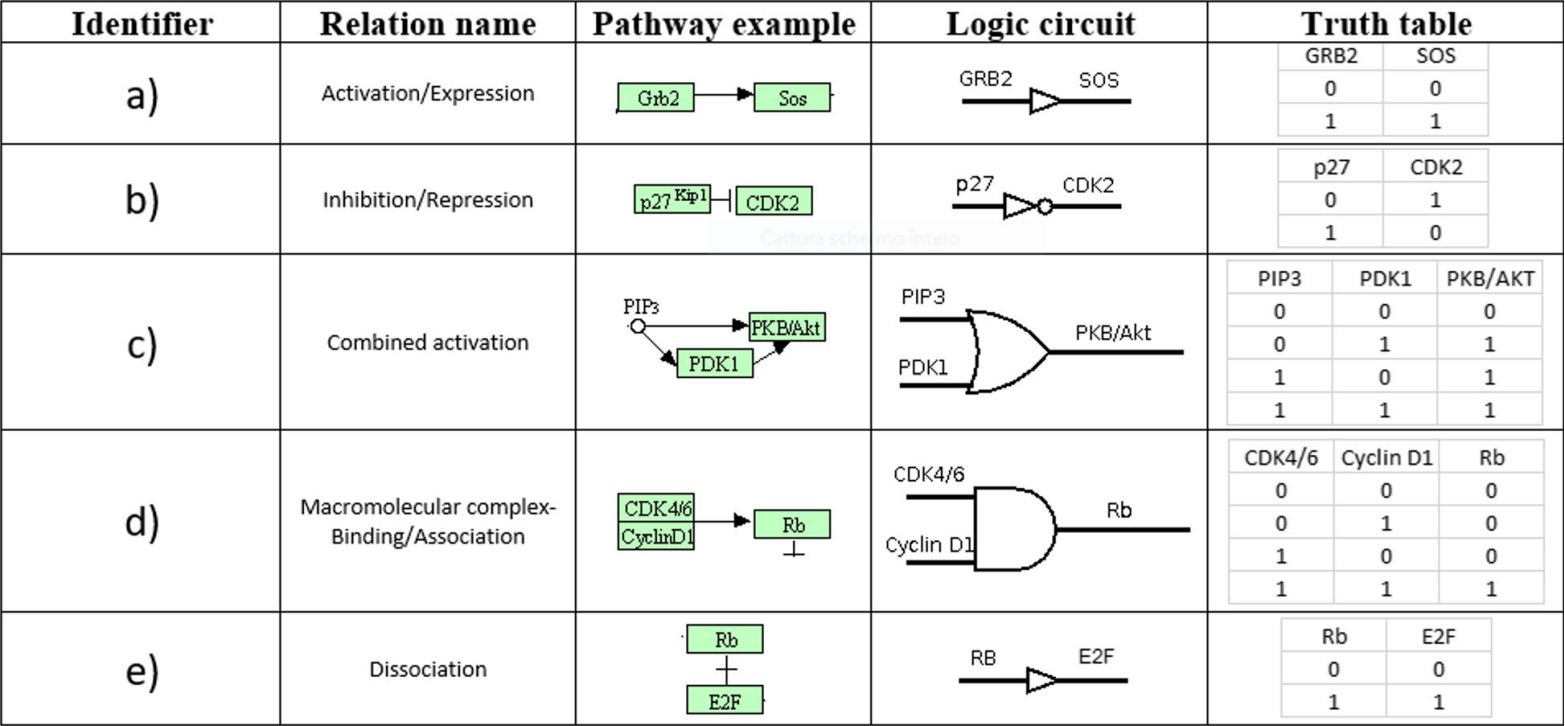
KEGG pathway logic modelling. In **a** the activation/expression relationship is modelled by a logic buffer gate. In **b** the inhibition/repression relationship is modelled by a logic NOT gate. In **c** the combined activation is modelled by a logic OR gate. In **d** the macromolecular complex activation and the binding/association relationship is modelled by a logic AND gate. In **e** the dissociation relationship is modelled by a buffer gate

The biological signalling pathway is converted to a digital circuit, made up of logic gates as BUFFER, NOT, OR and AND gates. Each signal corresponding to a gene in the pathway is considered as a Boolean variable. The value of the variable is 1 if the gene is expressed, 0 otherwise. Several input combinations can lead to different output combinations. In literature, some transformation rules from the biological pathway to the digital circuit and drug modelling are given [14]. The whole set of transformation rules is reported (Fig. 3): each row reports the equivalence between the KEGG relationship, on the left, and the corresponding logic gate with its truth table, both on the right.

According to KEGG database components, the modelled relationships are: activation/expression (Fig. 3a); inhibition/repression (Fig. 3b); combined activation (Fig. 3c); macromolecular complex and binding/association (Fig. 3d) and dissociation (Fig. 3e).

The activation relationship (Fig. 3a) is represented by a logic BUFFER gate, meaning that if the input gene is expressed, the output gene will be expressed. Otherwise, the output gene will not be expressed. The repression relationship (Fig. 3b) is represented by a logic NOT gate, meaning that if the input gene is expressed, the output gene will not be expressed and vice versa. A combined activation relationship (Fig. 3c) is represented by a logic OR gate, meaning that almost one among the input genes must be expressed to express the output gene.

According to the literature, in this paper we model the macromolecular complex (Fig. 3d) by an AND logic gate [14], meaning that both genes must be simultaneously expressed to express the output gene.

Binding/association relationships are modelled by a logic AND gate, as well as macromolecular complex. In fact, all bounded elements should be activated or expressed to activate/express or inhibit/repress the downstream proteins, genes or enzymes. KGML code clearly shows that binding/association relationships are introduced with reference to groups entries; therefore, the biological meaning of the binding/association relationship is the same of biological groups (macromolecular complexes). For this reason, we choose to model the groups as unique biological entities no matter which is the relation that creates these macromolecular complexes.

The relationship of dissociation (Fig. 3e) is considered as an activation as well. Indeed, in a macromolecular complex, a protein inhibits the other. In order to activate the latter protein, the macromolecular complex should be dissociated. As an example from KEGG database, the macromolecular complex is made up of Rb and E2F proteins (Fig. 3e). When they are bound, Rb inhibits E2F. In the pathway, Rb protein releases and consequently activates E2F, which leads to cell cycle progression (G1/S progression).

In the proposed structure of the circuit, we decided to not represent molecular events involved in the activation or deactivation of proteins, as phosphorylation. Indeed, these modifications do not affect the circuit relationships and logic gates. In fact, logic gates account for expression or inhibition of molecular species, not considering how inhibition or expression is achieved.

Missing interaction relationship, which is for example represented in KEGG database as an arrow with a sidelong segment, is not accounted for in the pathway logic circuit because the corresponding genes are not related to each other and consequently do not alter the final results.

Indirect activation (represented in a KEGG pathway as a dashed arrow) means that other intermediate genes, which are not included in the pathway, are involved. Indirect activation is considered as well as direct activation in order to not increase the complexity of the corresponding pathway since it does not alter the circuit functionality and final results.

**Fig. 4:**
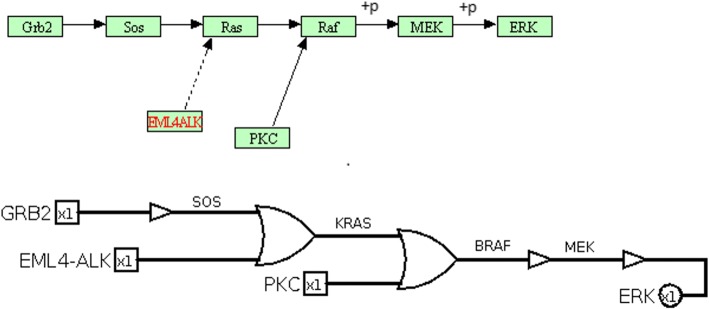
Simplified pathway model. At the top, a detail of KEGG non-small cell lung cancer pathway is shown. At the bottom, the corresponding digital circuit is shown. SOS gene is activated by GRB2 and therefore a buffer gate is used. KRAS and BRAF genes are activated by two genes and therefore two OR gates are used. Finally, MEK is activated by BRAF and ERK by MEK gene and two buffer gates are used

As an example, a detail of the non-small cell lung cancer (see Case study 1) KEGG pathway and its corresponding circuit are shown (Fig. 4).

Notice that each gene reported into the graphical representation of a KEGG pathway can represent a family instance; for example RAS corresponds to the KRAS gene and RAF to the BRAF gene. Of course, metadata reported in KEGG markup language (KGML) specifies the gene which is effectively involved in the pathway. SOS gene is activated by GRB2 and therefore a buffer gate is used. KRAS and BRAF genes are activated by two genes and therefore two OR gates are used. Finally, MEK is activated by BRAF and ERK by MEK gene and two buffer gates are used.

### Simulation

The input and output of the signalling pathway are represented by two binary vectors whose size is equal to the number of input and output genes, respectively, where each bit is equal to 1 if the corresponding gene is expressed (active), 0 otherwise. In a cancer related signalling pathway, the inputs of the pathway are growth factors and tumor suppressors. The output of the signalling pathway consists of genes playing a key role in proliferation or apoptosis.

**Fig. 5:**
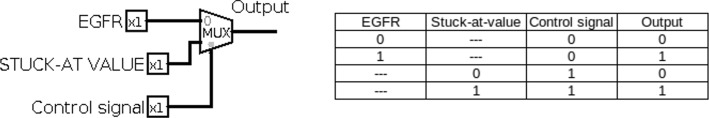
Mutations modelling. Multiplexer gate modelling the gene mutation and the corresponding truth table. If the control signal equals a logic value of 0, the input will be routed to the output. If the control signal equals a logic value of 1, the stuck-at value will be routed to the output

If all growth factors are set to 0 and tumor suppressors are set to 1, the output is non-proliferative, meaning that in this condition apoptosis without proliferation is involved. This is the best and less dangerous condition for the specific disease under study. Yet, because of gene mutations, even without growth factors and with active tumor suppressors, active proliferation with or without apoptosis could be obtained. Mutations are introduced as perturbations in the circuit, meaning that a fault in the Boolean network occurs every time a gene is mutated. Mutations are modelled by stuck-at-1 or stuck-at-0 faults [14]. Mutations of oncogenes are represented by stuck-at-1 faults, mutations of tumor suppressors by stuck-at-0 faults. The former consists of the disconnection of the faulted net from the upstream gates and the node is forced to a logic value of 1. The latter consists of the disconnection of the faulted net from the upstream gates and the node is forced to a logic value of 0. In order to study the influence of multiple gene mutations, in the same circuit these are applied by using a multiplexer component. With the aid of the multiplexer gate, the circuit could be automatically driven by the so-called control signal. The multiplexer gate features one output and multiple inputs, two in this case. For example (Fig. 5), EGFR and stuck-at value (logic value of 1 in this case) are the two inputs. A control signal managing the inputs is included. The multiplexer aims at connecting the output to one and one only input, according to the value of the control signal. In this case, two inputs are included and therefore the control signal is a binary signal assuming a logic 1 or logic 0 value. The control signal is an artificial signal, driven by the user to apply the corresponding mutation. If the multiplexer control signal is equal to 0, the input will be routed to the output and thus no mutation is active. If the multiplexer control signal is equal to 1, the stuck-at value will be routed to the output, thus disconnecting the upstream gates from the downstream gates of the logic circuit and consequently implementing the corresponding mutation.

A test vector is defined to enumerate and analyse the effect of all involved faults (mutations) in the Boolean network. The input test vector is identified by forcing to 0 each growth factor and forcing to 1 the tumor suppressors. If no growth factor is present and tumor suppressors are active, the output of the network corresponding to a healthy organism is non proliferative. By applying the input test vector and analysing the circuit under each mutation by observing the output vector, it is possible to check the level of proliferation caused by the mutation. During the simulation process, therefore, the input test vector represents a fixed input of the network.

**Fig. 6:**
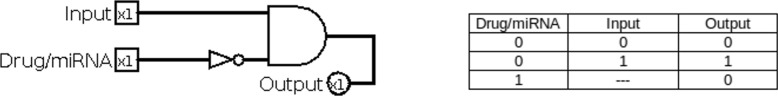
Drug or miRNA modelling. Drug or miRNA interaction model and the corresponding truth table.If drug or miRNA is applied (corresponding to a logic value of 1) the output gene is inhibited. Otherwise, the output equals the input, not affecting the circuit functionality

Drugs modify the circuit functionality altering the output of each mutation thus recovering faults and leading to less proliferative output. Effects of drugs can be analysed by the output of the digital circuit. The presence of drugs is modelled by using a drug vector with values set to 1 if the corresponding drug is applied to the circuit, 0 otherwise. In the digital circuit, drugs are properly introduced following the literature approach [14]. Drugs are introduced in the circuit by a combination of NOT and AND gates (Fig. 6), meaning that if drug is applied (corresponding to a logic value of 1) the output gene is inhibited [14]. In fact, the output of the AND gate follows the input if the drug is 0 (due to the inversion of the NOT gate).

For the fist time, in this work we introduce miRNAs into a logic circuit of a pathway. MiRNAs are modelled as well as drugs and are inserted in the circuit considering their gene targets. Once again, given the test vector, it is possible to observe the corresponding output affected by the mutations in the network. By analysing the output vector, miRNAs role in fault-recovery can be studied, thus identifying the best miRNA for each mutation and the best combination of miRNAs for all involved mutations.

In order to measure the “pathogenic degree” of a specific output condition, we defined a proper score. In literature, authors proposed a score based on the number of transcription factors and the number of active key proteins, considering on a non-linear many-to-one map [14]. In order to evaluate the effects of drugs and miRNAs on mutations, in this paper, a linear combination of proliferative and pro-apoptosis genes is proposed. The proposed score is quite easy to compute because it is a linear combination of output bits and allows biologists and clinicians to quantify the “pathogenic degree” of the output condition. The proposed score is obtained by counting the number of active proliferative genes and the number of active key pro-apoptosis genes and then computing the difference between them. The minimum score corresponds to the least proliferative output and condition. If the output is made up of seven bits, as given by:

1 $$ Output=[a,b,c,d,e,f,g]  $$

where *a*,*b*,*c*,*d* corresponds to proliferative genes and *e*,*f*,*g* corresponds to pro-apoptosis genes, the score is given by:

2 $$ S=P-A  $$

where P is the sum of the bits corresponding to proliferative genes, as given by:

3 $$ P=a+b+c+d  $$

and A corresponds to the sum of the last three bits corresponding to pro-apoptosis genes, as given by:

4 $$ A=e+f+g  $$

In this example, a possible output vector can be Output=[1010111]. In this case, the score is equal to S=P-A, where P=2 corresponding to the sum of the first four bits representing proliferative genes and A=3 representing the sum of the last three bits corresponding to the pro-apoptosis genes. In this example, the score is equal to S=-1. The most proliferative condition corresponds to Output=[1111000]. The score is equal to S=4 meaning cell proliferation without apoptosis. The less dangerous condition corresponds to Output=[0000111], whose score is equal to S=-3, corresponding to absence of proliferation with apoptosis. The scores S=4 and S=-3 are unique, each corresponding to a specific configuration of output bits. Other scores cannot be associated to a specific bits configuration, but the aim is at identifying dangerous conditions no matter of which specific gene is expressed or not, but only considering their role and the number of active genes.

A cumulative score is assigned to each drug or miRNA by summing the scores of each mutated output under the effect of the drug or miRNA under study. The most effective drug or miRNA corresponds to the minimum cumulative score.

### Flowchart of the proposed method

**Fig. 7:**
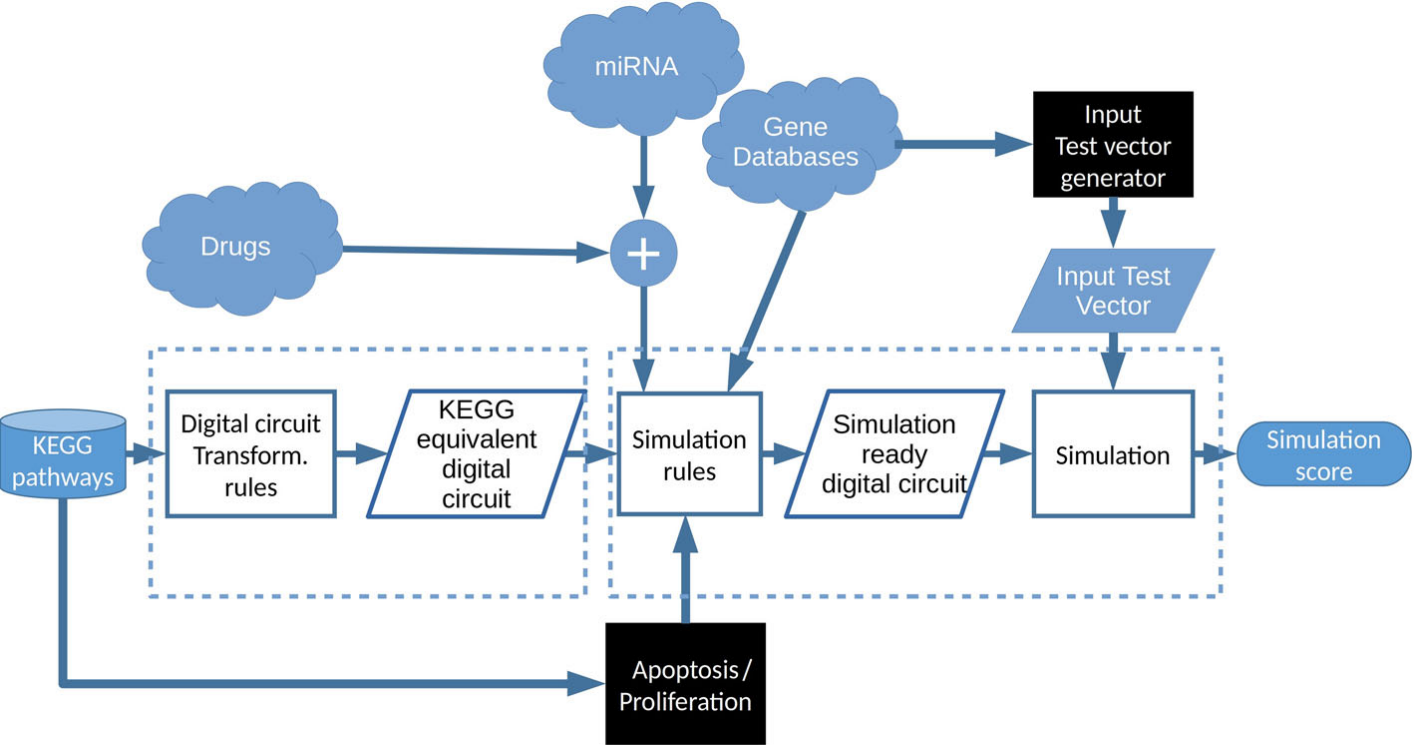
Flowchart of the proposed model. From the KEGG pathway database, the user selects a cancer pathway and by digital circuit transformation rules, the equivalent circuit of the selected pathway is obtained. By applying simulation rules, drugs or miRNAs are introduced in the circuit as well as gene mutations, according to the explanation given in the “Simulation” section. Looking at the KEGG pathway, we identify pro-apoptotic and proliferative genes. Therefore, the simulation ready circuit is ready. After applying the input test vector, simulation is running thus resulting in the simulation score

**Fig. 8:**
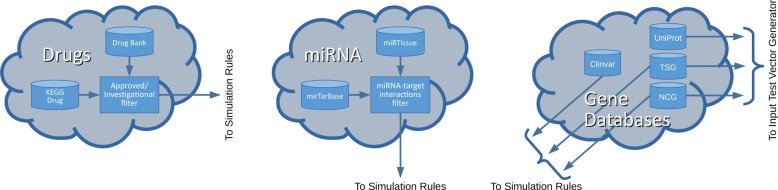
A detail of the proposed flowchart. On the left “cloud” component drug databases are explained: Drugs are selected from KEGG drug and DrugBank databases and then filtered by approved or investigational status. In the middle “cloud” component, miRNAs selection is explained: miRNAs are selected from miRTarBase and miRTissue databases: post-filtering occurs as discussed in the “Simulation” section. In the right “cloud” the gene databases are explained: mutations are retrieved from ClinVar and applied to simulation rules as well as information about tumor suppressive or oncogenic role of mutated genes, retrieved from TSG and NCG databases; in order to generate the input test vector, growth factors (retrieved from UniProt), tumor suppressors and oncogenes are identified

In this Section, we summarize the methods described in the previous subsections by means of a flowchart (Fig. 7). The figure includes two dashed boxes. On the left, the dashed box shows the processing steps adopted to transform the selected pathway into an equivalent digital circuit, as described in the “Logic circuit of the pathway” Section. On the right, the dashed box reflects the processing steps described in the “Simulation” Section. Cylinders stand for databases, rectangular blocks represent processing steps, parallelograms are input or output data of other rectangles, black rectangles are manually curated processing steps, “cloud” components represent small subcharts (Fig. 8).

Starting from the selected pathway from KEGG pathway database, the transformation rules described in the “Logic circuit of the pathway” section are applied. This way, we obtain the equivalent digital circuit, corresponding to the selected pathway. Then, we can introduce drugs or miRNAs in the circuit, as described in the “Simulation” section.

More in detail (Fig. 8), as described in the “Materials” section, drugs are retrieved from KEGG drug and DrugBank databases. The user filters out the drug list based on approved/investigational status of the drug itself. miRNAs are retrieved from miRTarBase and miRTissue databases as described in the “Simulation” section. miRNAs post-filtering occurs to avoid circuit redundancy.

Simulation rules include the modelling of gene mutations, drugs and miRNAs, according to the “Simulation” section. Simulation ready digital circuit includes, therefore, the mutations, drugs or miRNAs. More in detail, in Gene Databases subchart (Fig. 8) we can see how the gene mutations are retrieved from ClinVar database and tumor suppressors or oncogenes are identified by TSG and NCG databases in order to identify stuck-at-1 and stuck-at-0 faults. Info about about pro-apoptotic or proliferation features of the output genes are manually extracted from KEGG pathway. As previously described in the “Simulation” section, the input test vector is identified and applied to the simulation ready digital circuit before performing the simulation. Growth factors and tumor suppressors are retrieved from databases, as described in the “Materials” section and the user generates the input test vector by fixing at 1 the tumor suppressors and at 0 the growth factors. After running the simulation, the system outputs the simulation scores.

## Results and discussion

In this Section, we present two different case studies in order to prove that our method can be successfully applied to different cancer disease pathways. Case study 1 deals with non-small cell lung cancer (NSCLC); case study 2 is about melanoma. For each scenario, firstly we introduce a brief description of the selected pathology, and then we present the circuit design and the simulation setup; finally, we report and discuss the obtained results for the drug discovery simulation and the miRNA therapeutics simulation, respectively.

### Case study 1: non-small cell lung cancer

The non-small cell lung cancer represents the 80% of lung cancers. Molecular mechanism governing lung cancer pathogenesis and progression has been the focus of many research works during the last years and these efforts aim at developing new approaches based on targeted therapies. This is in accordance with the progressive discovery of different mutations in several genes involved in different biological processes regulating lung cancer development and progression. As an example, epidermal growth factor receptor (EGFR) [29], anaplastyc lymphoma kinase (ALK) [30], and Kirsten rat sarcoma viral oncogene homolog (K-RAS) [31] mutations, are considered prognostic and predictive biomarkers and targets for personalized drugs [32, 33]. In this context, the use of miRNA therapeutics as “next generation targeted drugs” seems to be promising [34]. Indeed, some in vitro and in vivo preclinical studies began to investigate the use of specific miRNAs in assisting various chemotherapeutic strategies of intervention in NSCLC [35]. We decided to test our logic circuit on this cancer type because different pathogenic mutations are reported from the literature in different genes belonging to different cellular pathways, and there is a good knowledge of the cellular steps governing lung cancer pathogenesis and progression. As example MAPK signalling pathway is a well known signalling cascade in which different genes can be mutated in NSCLC. 11 different gene mutations were inserted in our circuit (Table 1). For drug discovery analysis, 6 different drugs (Table 2) were selected.

**Table 2:** NSCLC case study: Drugs, left column, and their corresponding targets, right column, related to the proposed case study

Drug	Target
Osimertinib	EGFR
Alectinib	ALK
Dabrafenib	BRAF
Trametinib	BRAF,MEK
Everolimus	AKT
BKM120	PI3K

#### NSCLC circuit design and simulation

**Fig. 9:**
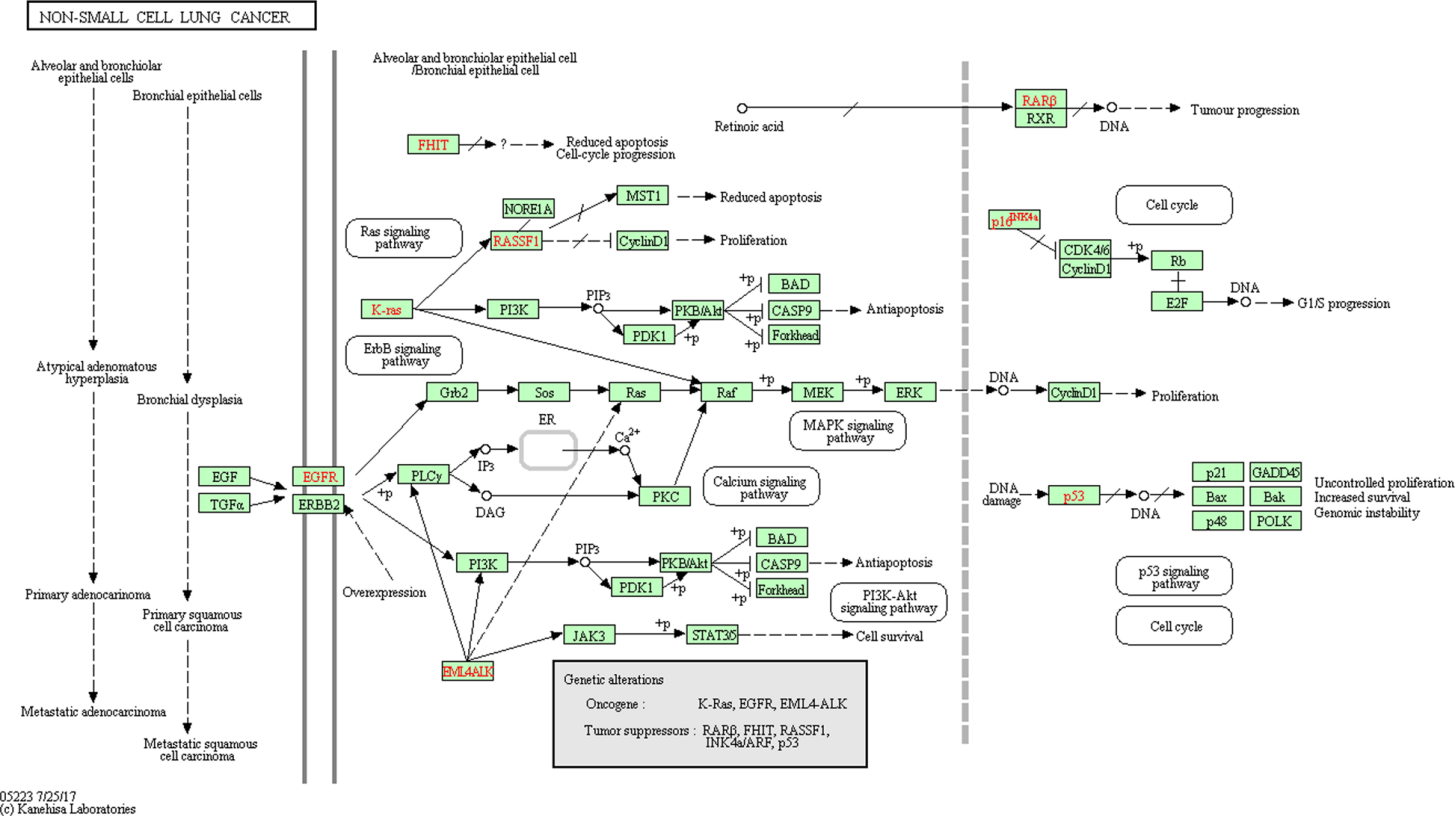
Non small cell lung cancer KEGG pathway. The KEGG non-small cell lung cancer pathway is shown. The figure shows all KEGG relationships of activation, inhibition, combined activation and macromolecular complex activation. Indirect and missing interaction are also shown

NCLSC KEGG pathway (Fig. 9) is the starting point in order to build its related digital circuit. Inputs are three growth factors, inducing proliferation, EGF, TGA and EML4-ALK and two tumor suppressors, p16 and PTEN. There are seven key genes as outputs: four of them induce proliferation (i.e. CCND1, E2F, MST1, STAT3/5) and the other three are pro-apoptosis genes (i.e. BAD, CASP9 and FORKHEAD, FORKHEAD is also known as FOXO3).

**Fig. 10:**
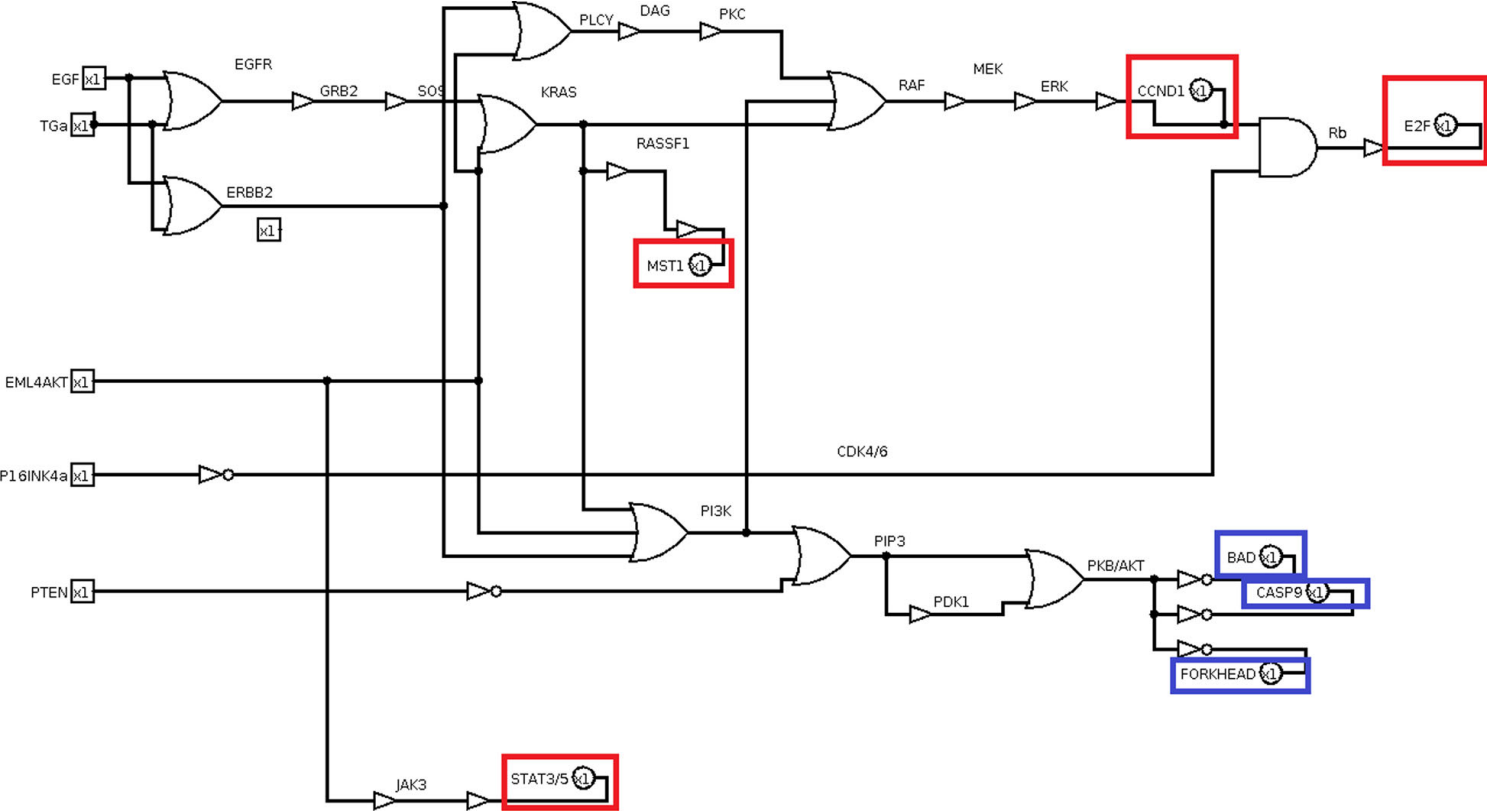
Non small cell lung cancer pathway circuit. In red boxes proliferative outputs and in blue boxes pro-apoptosis outputs are included

Closely following the transformation rules (Fig. 3), the equivalent circuit of the whole KEGG non-small cell lung cancer pathway is obtained (Fig. 10, red and blue boxes identify proliferative and pro-apoptosis genes, respectively). The logic circuits implementation and simulation are carried out by Logisim software [36].

The logic circuit is analysed by observing the output vector under each mutation and a specific input test vector. In this case, the test vector is made up of five bits that represent the repression of three growth factors and the activation of two tumor suppressors, [EGF,TGA,EML4-ALK, p16,PTEN], where growth factors are set to 0 and tumor suppressors are set to 1. The test vector is then equal to [00011]. The output vector is [CCND1, E2F, MST1, STAT3/5, BAD, CASP9, FORKHEAD]. The test vector generates a non-proliferative output with apoptosis, which is equal to [0000111], corresponding to a score S=-3. Correctly, if no growth factor is present and tumor suppressors are active, the output is non proliferative with apoptosis.

Because of mutations, the circuit is altered in its functionality, thus eventually leading to proliferative output or inhibition of pro-apoptosis genes even with no growth factor and active tumor suppressors. The input test sequence [00011] allows testing all faults caused by mutations.

**Fig. 11:**
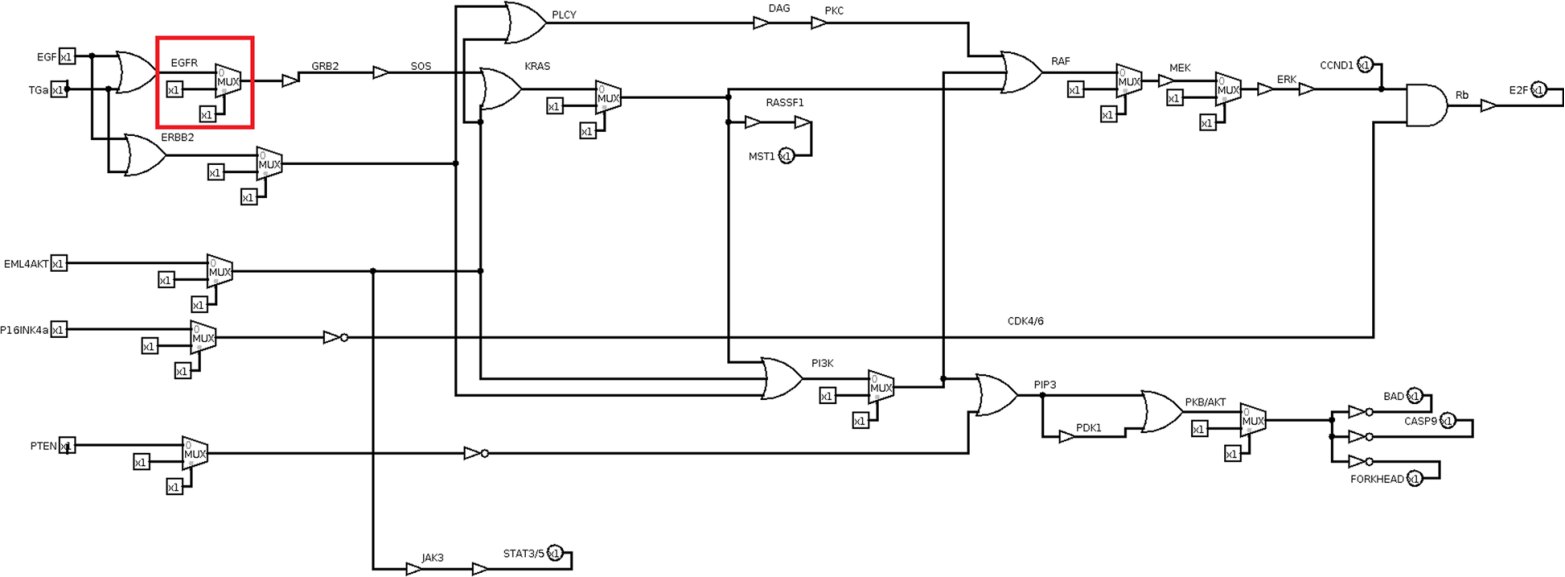
Non small cell lung cancer pathway circuit with mutations. Mutations are modelled by multiplexer logic gates. EGFR mutated model is included in the red box

The logic circuit is modified accounting for eleven mutations (Table 1), introducing the multiplexer gates (Fig. 11).

For this case study, the score or ranking is calculated by summing the first four bits (proliferative genes) and subtracting the last three bits (pro-apoptosis genes). The least score corresponds to the least dangerous output. For example, the non proliferative output [0000111] is assigned to a score of S=-3, corresponding to a non proliferative output with apoptosis. The best score is consequently S=-3. The most proliferative output corresponds to [1111000], associated to a score of S=4, meaning that proliferation without apoptosis is obtained.

#### NSCLC drug discovery simulation results

For the case study, drugs of interest are obtained from KEGG drugs and DrugBank databases (Table 2).

The drug we analyse are: Osimertinib which targets EGFR gene, Alectinib which targets ALK gene, Dabrafenib which targets BRAF gene, Trametinib which targets BRAF and MEK genes, Everolimus which targets AKT gene and BKM120 which targets PI3K gene. Then, the drug vector is [Osimertinib, Alectinib, Dabrafenib, Trametinib, Everolimus, BKM120].

**Fig. 12:**
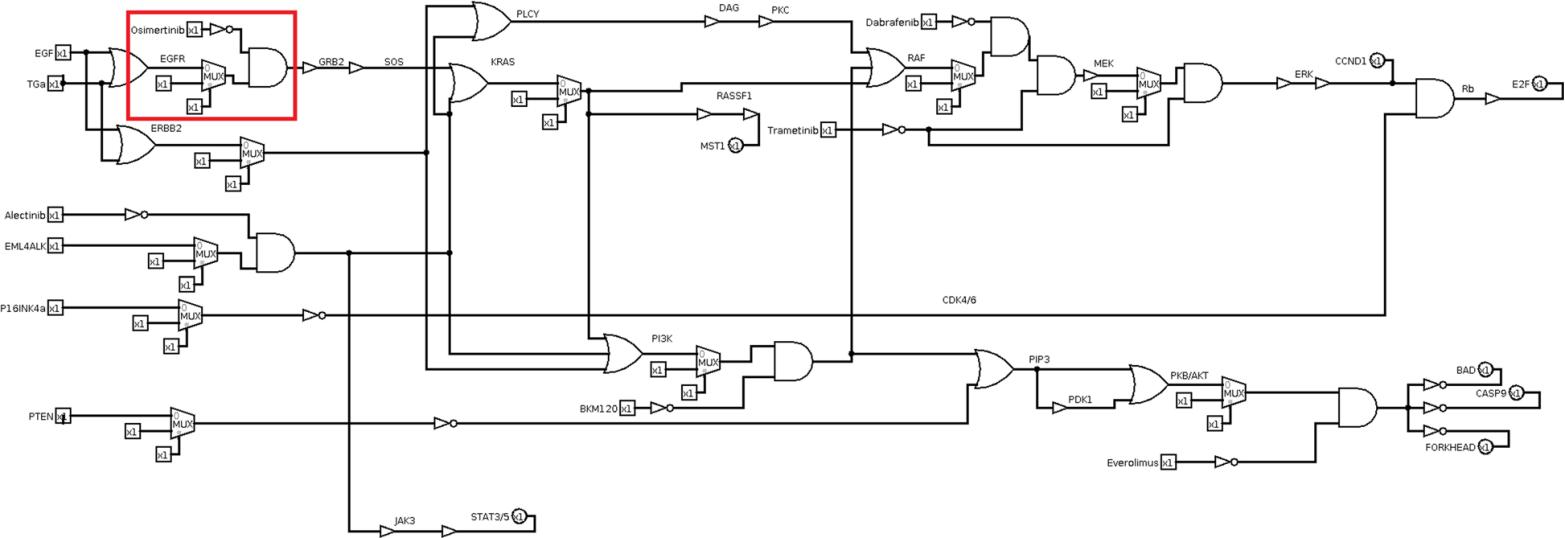
Non small cell lung cancer pathway circuit with mutations and drugs. Drugs are modelled by a logic NOT and a logic AND gates. EGFR mutated model is included in the red box

In the corresponding logic circuit with drugs (Fig. 12), the part corresponding to the EGFR mutated cancer is included in the red box, corresponding to the first row of Table 1. The drug action is applied to the output of the multiplexer stage, that represents the mutation. In fact, Osimertinib targets EGFR. Consequently, if Osimertinib is active, it inhibits the EGFR gene. Whatever the output of the multiplexer is, i.e. gene mutated or not, the drug inhibits the EGFR gene. If Osimertinib is not active, the downstream gates are connected to the multiplexer output. Then, if the control signal is zero, the downstream gates are connected to the EGFR gene, otherwise the downstream gates are connected to the logic stuck-at value of 1.

Given a fixed input representing the test vector, the circuit is simulated in order to find the best drug for each mutation and the best combination of two drugs for all involved mutations. Combinations with no more than two drugs are accounted for, in order to avoid considerable side-effects.

We computed the effect of each drug on each mutation (Table 3). For each mutation on the rows and for each drug on the columns, the scores of the faulted output without and with drug are shown, separated by an arrow. The best drug generates the most non proliferative output (lowest ranking) for the selected mutation. As an example, the best drug for EGFR mutated non small cell lung cancer is Osimertinib. Among selected drugs, Everolimus is one of the most effective drugs because it acts on all mutations except two, with two rectified faults for which the output is equal to the fault-free output.

**Table 3:** NSCLC case study. Effects of drugs, in terms of the score defined in “Simulation” Section, on gene mutations

Drugs	EGFR	ERBB2	EML4-ALK	PTEN	KRAS	BRAF	PIK3CA	AKT	MEK	EML4-ALK+p16	Ranking
Score obtained by a single drug											
Everolimus	2 → -1	1 → -2	3 → 0	0 → -3	2 → -1	-2 → -2	1 → -2	0 → -3	-2 → -2	4 → 1	-15
BKM120	2 → -1	1 → -2	3 → 0	0 → 0	2 → -1	-2 → -2	1 → -3	0 → 0	-2 → -2	4 → 1	-10
Alectinib	2 → 2	1 → 1	3 → -3	0 → 0	2 → 2	-2 → -2	1 → 1	0 → 0	-2 → -2	4 → -3	-4
Trametinib	2 → 1	1 → 0	3 → 2	0 → 0	2 → 1	-2 → -3	1 → 0	0 → 0	-2 → -3	4 → 2	0
Dabrafenib	2 → 1	1 → 0	3 → 2	0 → 0	2 → 1	-2 → -3	1 → 0	0 → 0	-2 → -2	4 → 2	1
Osimertinib	2 → -3	1 → 1	3 → 3	0 → 0	2 → 2	-2 → -2	1 → 1	0 → 0	-2 → -2	4 → 4	4
Score obtained by the combination of two drugs											
Everolimus	2 → -1	1 → -3	3 → -1	0 → -3	2 → -2	-2 → -3	1 → -3	0 → -3	-2 → -3	4 → -1	-23

Combinations of two drugs are tested. The best combination consists of Everolimus/Trametinib (Table 4). For each mutation (first-column), faulted output without drugs (second column), faulted output under Everolimus/Trametinib (third column) and faulted with and without drug ranking, separated by an arrow,(fourth column) are shown. The analysed combination is the best among tested. Everolimus/Trametinib acts on each mutation and six absolutely non proliferative outputs are achieved (minimum ranking S=-3). Six faults are completely rectified to fault-free output. In five mutations (EGFR, EML4-ALK, KRAS,PI3K,EML4-ALK+p16), the action of combined Everolimus/Trametinib is stronger than single drugs Everolimus or Trametinib. These theoretical results are confirmed in literature, where that combination is tested on 67 patients in phase IB [37]. The study is reported on clinicaltrials.gov. Yet, the study does not proceed to phase II since adequate dose providing an acceptable tolerability and drug exposure was not found. However, the combination has been tested, thus validating the proposed digital approach.

**Table 4:** A detailed view of effects of Everolimus/Trametinib on gene mutations, highlighting the circuit output without (second column) and with drug (third column), and the corresponding change in the score

Mutation	Faulted output	Faulted output	Score change
	without drug	with drug	
EGFR	[1010000]	[0010111]	2 →-1
ERBB2	[1000000]	[0000111]	1 →-3
EML4-ALK	[1011000]	[0011111]	3 →-1
PTEN	[0000000]	[0000111]	0 →-3
KRAS	[1010000]	[0010111]	2 →-2
BRAF	[1000111]	[0000111]	-2 →-3
PIK3CA	[1000000]	[0000111]	1 →-3
AKT	[0000000]	[0000111]	0 →-3
MEK	[1000111]	[0000111]	-2 →-3
EML4-ALK+p16	[1111000]	[0011111]	4 →-1

#### NSCLC miRNA therapeutics simulation results

miRNAs are expressed in normal tissues. In case of cancer, some miRNAs are found to be over- or under-expressed. miRNA targets which are involved in the biological pathway of the specific cancer disease can be thus over- or under-expressed, leading to proliferation or anti-apoptosis.

As introduced in the “Materials” subsection, we retrieve tissue specific validated miRNA-target interactions by miRTissue tool. Lung adenocarcinoma is selected as tissue specific. Degradation is selected as interaction type and all genes involved in the non small cell lung cancer are introduced in the search box. All involved miRNAs are then filtered by a p-value less than 0.05 and by targets. As a result, we obtained 106 miRNA-target interactions.

Among those interactions, we select the corresponding miRNAs addressing multiple targets as well as miRNAs targeting a single gene not covered by other selected miRNAs. As a result, 38 miRNAs are selected.

Some miRNAs share exactly the same targets. Since from a circuit perspective miRNAs sharing the same targets have the same effect on the circuit functionality, we consider a representative miRNA for each group to avoid circuit redundancy. The representative is chosen as the most common in the literature in order to perform a comparison with literature results. As a result of the post-filtering process, 20 miRNAs are obtained (Table 5).

**Table 5:** NSCLC case study. miRNAs and their targets selected for the proposed case study

miRNA	Target
let-7c	CCND1, KRAS
let-7g	AKT,CCND1
mir-130b	RASSF1,STAT3
mir-142	CCND1,FOXO3, ERK
mir-15a	AKT, CCND1
mir-17	PKC, STAT3
mir-18a	CCND1,TGFA
mir-191	BRAF
mir-193b	CCND1, PKC
mir-27a	SOS
mir-27b	PDK1
mir-29a	AKT,CDK4
mir-30e	KRAS,TGA
mir-330	BAD
mir-335	FOXO3,JAK3
mir-34a	CDK6,MEK
mir-375	PI3K,PKC
mir-4778	PKC, STAT/3
mir-4789	CCND1, STAT5
mir-497	CDK4, GRB2

**Fig. 13:**
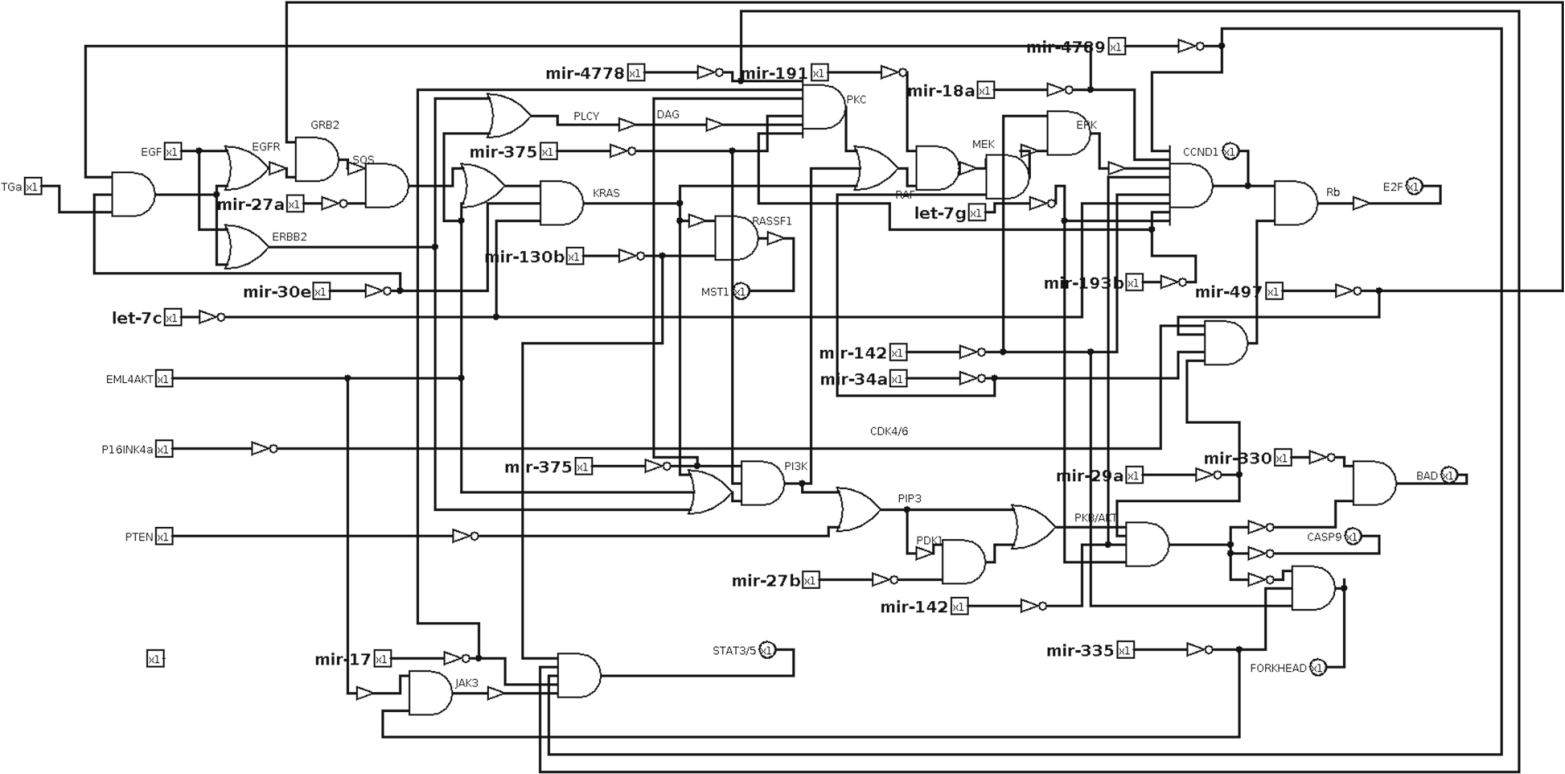
Non small cell lung cancer pathway logic circuit with miRNA. miRNAs are modelled as well as drugs by a logic NOT and a logic AND gates

The circuit is modified to introduce miRNAs replacing drugs (Fig. 13). The circuit is analysed to identify the best miRNA for each mutation and the best combination of two miRNAs for all involved mutations.

Simulation results are shown (Table 6). In the last column, a cumulative ranking is introduced. The cumulative ranking is obtained by summing the ranking on each row. Each miRNA corresponds to a cumulative ranking. The minimum cumulative ranking corresponds to the most effective miRNA. The best combination is let-7c/let-7g corresponding to a cumulative ranking of -27. The best single miRNA is let-7g corresponding to a cumulative ranking of -24. As an example, miR-330 and miR-335 worsen the output of two mutations, thus they should be treated with antimiR.

**Table 6:** NSCLC case study. Network scores obtained considering miRNAs acting on mutated gene targets. The table is organized considering the score obtained by a single miRNA, by a pair of miRNAs and by three miRNA, respectively. The last column, called ranking, represents the sum of the scores of a miRNA, or combination of miRNAs, corresponding to each gene target

miRNA	EGFR	ERBB2	EML4-ALK	PTEN	KRAS	BRAF	PIK3CA	AKT	MEK	EML4-ALK+p16	Ranking
Score obtained by a single miRNA											
let-7g	2 → -2	1 → -3	3 → -1	0 → -3	2 → -2	-2 → -3	1 → -3	0 → -3	-2 → -3	4 → -1	-24
mir-15a	2 → -2	1 → -3	3 → -1	0 → -3	2 → -2	-2 → -3	1 → -3	0 → -3	-2 → -3	4 → -1	-24
mir-29a	2 → -2	1 → -2	3 → 0	0 → -3	2 → -1	-2 → -2	1 → -2	0 → -3	-2 → -2	4 → 0	-13
let-7c	2 → -3	1 → 0	3 → 1	0 → 0	2 → 1	-2 → -3	1 → 0	0 → 0	-2 → -3	4 → 1	-6
mir-375	2 → -1	1 → -3	3 → 0	0 → 0	2 → -1	-2 → -2	1 → 1	0 → 0	-2 → -2	4 → 1	-4
mir-27a	2 → -3	1 → 1	3 → 3	0 → 0	2 → 2	-2 → -2	1 → 1	0 → 0	-2 → -2	4 → 4	-3
mir-4789	2 → 1	1 → 0	3 → 1	0 → 0	2 → 1	-2 → -3	1 → 0	0 → 0	-2 → -3	4 → 1	-2
mir-330	2 → 2	1 → 1	3 → 3	0 → 0	2 → 2	-2 → -1	1 → 1	0 → 0	-2 → -1	4 → 4	-2
mir-497	2 → -3	1 → 1	3 → 3	0 → 0	2 → 2	-2 → -2	1 → 1	0 → 0	-2 → -2	4 → 3	0
mir-18a	2 → 1	1 → 0	3 → 2	0 → 0	2 → 1	-2 → -3	1 → 0	0 → 0	-2 → -3	4 → 2	0
mir-193b	2 → 1	1 → 0	3 → 2	0 → 0	2 → 1	-2 → -3	1 → 0	0 → 0	-2 → -3	4 → 2	0
mir-34a	2 → 1	1 → 0	3 → 2	0 → 0	2 → 1	-2 → -3	1 → 1	0 → 0	-2 → -3	4 → 2	0
mir-27b	2 → 2	1 → 1	3 → 3	0 → 0	2 → 2	-2 → -2	1 → 1	0 → 0	-2 → -2	4 → 4	0
mir-142	2 → 1	1 → 0	3 → 2	0 → 0	2 → 1	-2 → -2	1 → 0	0 → 0	-2 → -2	4 → 2	2
mir-30e	2 → -3	1 → 1	3 → 2	0 → 0	2 → 2	-2 → -2	1 → 1	0 → 0	-2 → -2	4 → 3	2
mir-191	2 → 1	1 → 0	3 → 2	0 → 0	2 → 1	-2 → -3	1 → 0	0 → 0	-2 → -2	4 → 2	3
mir-335	2 → 2	1 → 1	3 → 2	0 → 0	2 → 2	-2 → -1	1 → 1	0 → 0	-2 → -1	4 → 3	3
mir-17	2 → 2	1 → 1	3 → 2	0 → 0	2 → 2	-2 → -2	1 → 1	0 → 0	-2 → -2	4 → 3	5
mir-130b	2 → 1	1 → 1	3 → 1	0 → 0	2 → 1	-2 → -2	1 → 1	0 → 0	-2 → -2	4 → 2	5
mir-4778	2 → 2	1 → 1	3 → 2	0 → 0	2 → 2	-2 → -2	1 → 1	0 → 0	-2 → -2	4 → 3	5
Score obtained by the combination of two miRNAs											
let-7c/let-7g	2 → -3	1 → -3	3 → -2	0 → -3	2 → -2	-2 → -3	1 → -3	0 → -3	-2 → -3	4 → -2	-27
let-7g/mir-17	2 → -2	1 → -3	3 → -2	0 → -3	2 → -2	-2 → -3	1 → -3	0 → -3	-2 → -3	4 → -2	-26
let-7g/mir-20a	2 → -2	1 → -3	3 → -1	0 → -3	2 → -2	-2 → -3	1 → -3	0 → -3	-2 → -3	4 → -1	-24
let-7g/miR-335	2 → -1	1 → -2	3 → -1	0 → -2	2 → -1	-2 → -2	1 → -2	0 → -2	-2 → -2	4 → -1	-16
mir-20a/mir-335	2 → 2	1 → 1	3 → 2	0 → 0	2 → 2	-2 → -1	1 → 1	0 → 0	-2 → -1	4 → 3	3
mir-335/mir-17	2 → 2	1 → 1	3 → 2	0 → 0	2 → 2	-2 → -1	1 → 1	0 → 0	-2 → -1	4 → 3	3
mir-20a/mir-17	2 → 2	1 → 1	3 → 2	0 → 0	2 → 2	-2 → -2	1 → 1	0 → 0	-2 → -2	4 → 3	5
Score obtained by the combination of three miRNAs											
let-7g/mir-17/mir-20a	2 → -2	1 → -3	3 → -2	0 → -3	2 → -2	-2 → -3	1 → -3	0 → -3	-2 → -3	4 → -2	-26

Results are validated by the literature, where let-7 family is recognized as a potential therapeutic for murine lung cancer. It was analysed by injecting miRNAs by lentiviral vectors [38–44]. let-7 family is particularly efficient for KRAS mutated murine lung cancer in terms of reduction of the tumor formation [39]. In lung squamous cell carcinomas (subtype of the non-small cell lung cancer), low levels of let-7 are founded. let-7 plays a key role in tumor progression and formation, mainly through KRAS. In addition, The ectopic expression of let-7g in KRAS G12D mutated murine lung cancer induces both cell cycle arrest and cell death [40]. Inducing let-7g by lentiviral vectors, significant growth reduction of both human and murine non-small cell lung cancer is observed. let-7g mediated tumor suppression is more efficient in KRAS mutated tumors than with other mutations [40]. All these results confirm that miRNA mimics of the let-7 family is a potential treatment for the NSCLC and these results are confirmed by the circuit simulation. Furthermore, the proposed analysis approach highlights the efficiency of let-7 family mimics even for the other mutated genes. Our in silico experiments allow to highlight more specific results than the existing in vitro or in vivo experiments. While in the literature let-7 miRNA family is considered as a whole, the proposed circuit simulation identifies two specific miRNAs of the family; let-7c and let-7g together act as tumor suppressors in several mutated types of NSCLC, not only in KRAS mutated ones.

### Case study 2: melanoma

**Fig. 14:**
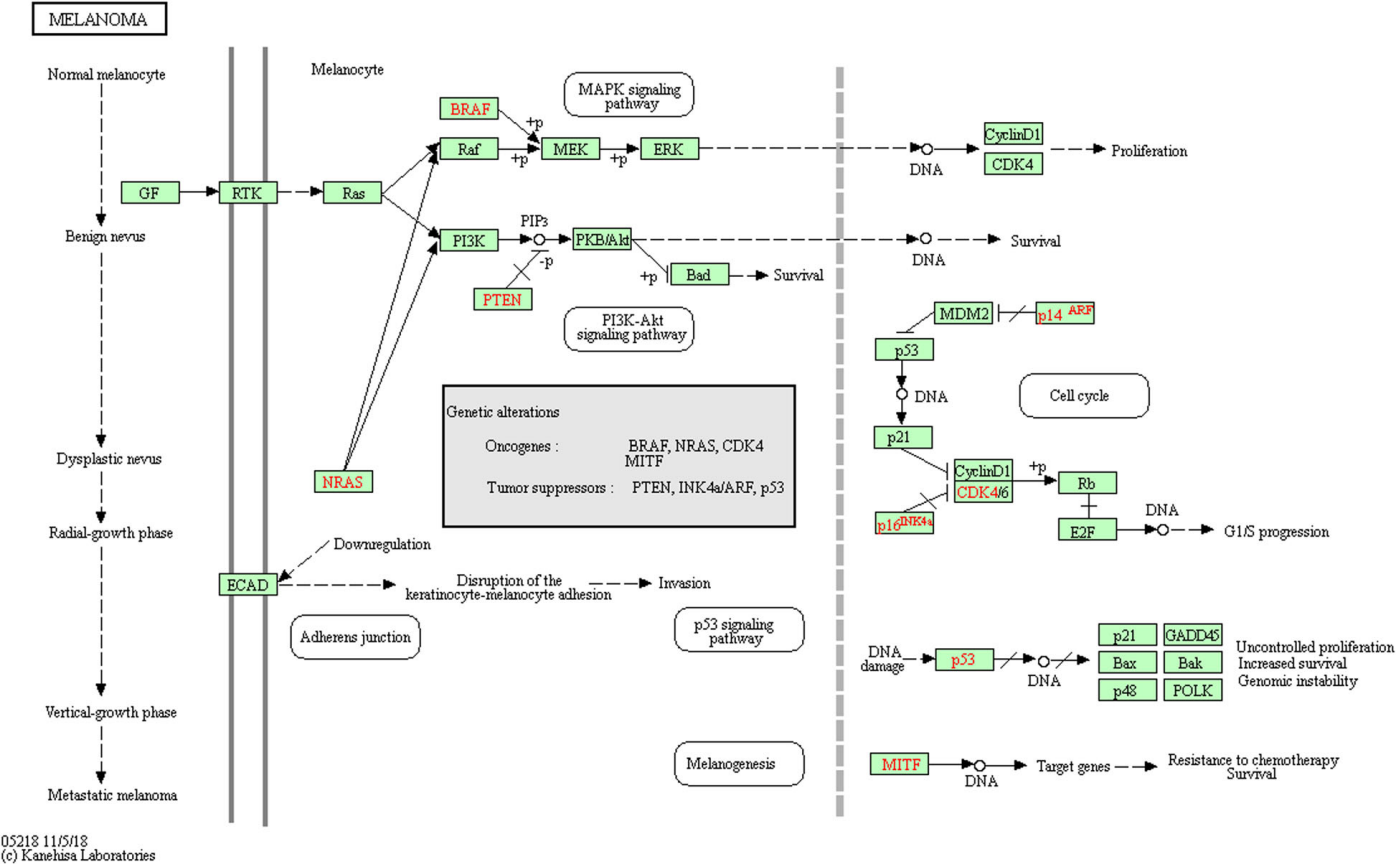
Melanoma KEGG pathway. The figure shows all KEGG relationships of activation, inhibition and combined activation involved in the melanoma pathway

Among cancer pathways in KEGG, we selected the melanoma disease as the second case study. Malignant melanoma is a highly aggressive tumor with high metastatic potential [45]. Although there are still many weaknesses in the comprehension of its pathogenesis, genetic studies have brought about many improvements in the last years. Indeed, the best oncogenes studied linked to this pathology are NRAS and BRAF mutations, both as prognostic factors and targeted therapeutic agents [46, 47]. Both NRAS and BRAF genes act via the mitogen-activated protein kinase (MAPK) signalling cascade, leading to the regulation of different cellular processes as proliferation, cell cycle progression, differentiation and apoptosis, and involve different other relevant proteins as cyclin D1, cyclin E, p21, p16, CDK4/6 (cyclin-dependent kinase 4/6) and others [48]. Dysregulation of any of these points of the pathway may also play a role in some cases of malignant melanomas [49]. PTEN is another relevant gene whose inactivating mutation can be associated with malignant phenotype in 30% of melanomas. Moreover, recent studies evidenced potential cooperation between PTEN and BRAF gene; for instance, authors associate the simultaneous presence of inactivating mutations in the former and activating mutations in the latter with the metastatic processes in melanoma mouse models [50]. The melanoma pathway retrieved from the KEGG database (Fig. 14) has two inputs: a growth factor (GF) and an oncogene (NRAS). Therefore the test vector is [GF,NRAS]=[0,0]. The output vector consists of three proliferative genes (E2F,CDK4/6,CCND1) and a pro-apoptotic gene (BAD). The output vector is [E2F, CDK4/6, CCND1, BAD]. By ClinVar, we selected eight mutations, filtering all genes in the targeted pathway by “melanoma” condition field (Table 7).

**Table 7:** Melanoma case study. Gene mutations retrieved from ClinVar. Gene name, accession number, mutation, clinical significance and literature references are reported, respectively

Gene	Accession	Mutation	Clinical significance	Ref
BRAF	NM_004333.4	c.1799T>G (p.Val600Gly)	Pathogenic	([74])
RAF(ARAF)	NM_001654.4	c.640T>G (p.Ser214Ala)	Likely pathogenic	([75])
NRAS	NM_002524.4	c.182_183delAAinsGG (p.Gln61Arg)	Pathogenic	([76])
RAS (KRAS)	NM_004985.4	c.351A>C (p.Lys117Asn)	Pathogenic/Likely pathogenic	([75])
AKT1	NM_005163.2	c.235C>A (p.Gln79Lys)	Likely pathogenic	([77])
PI3K	NM_006218.3	c.3140A>T (p.His1047Leu)	Pathogenic	([78])
MEK/MAP2K1	NM_002755.3	c.157T>C (p.Phe53Leu)	Pathogenic	([79])
CDK/CDKN2A	NM_000077.4	c.148C>T (p.Gln50Ter)	Pathogenic	([80])

#### Melanoma circuit design and simulation

**Fig. 15:**
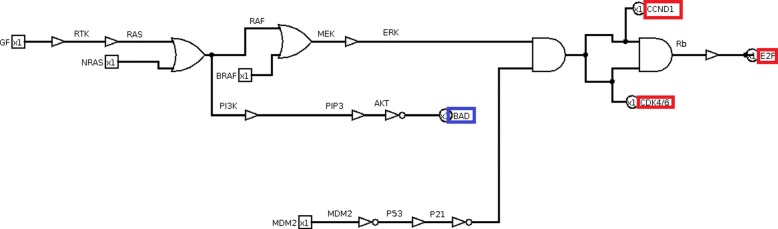
Melanoma cancer pathway digital circuit. The figure shows the equivalent digital circuit of the melanoma cancer pathway which has been retrieved from the KEGG pathway database. In red boxes proliferative output genes and in the blue box the pro-apoptotic output gene are included

We built the equivalent digital circuit for the melanoma case study (Fig. 15), obtained by following the given transformation rules (Fig. 3) applied to the melanoma pathway from KEGG database. Starting from a specific input test vector, we analyse the circuit by observing the output vector for each mutation. In this case study, the input vector consists of two bits [GF, NRAS], representing the inhibition of one growth factor and an oncogene. We define the fixed input test vector by forcing to 0 the growth factor and the oncogene. The input test vector is equal to [00]. The output vector is [E2F, CDK4/6, CCND1, BAD]. The input test vector generates a non-proliferative output with apoptosis, corresponding to the output vector [0001]. The associated score is equal to S=-1. Correctly, if no growth factor is active and the input oncogene is inhibited, the output is non-proliferative with apoptosis. Because of mutations, the circuit is altered in its functionality thus leading to proliferative output with eventually inhibited apoptosis.

#### Melanoma drug discovery simulation results

**Fig. 16:**
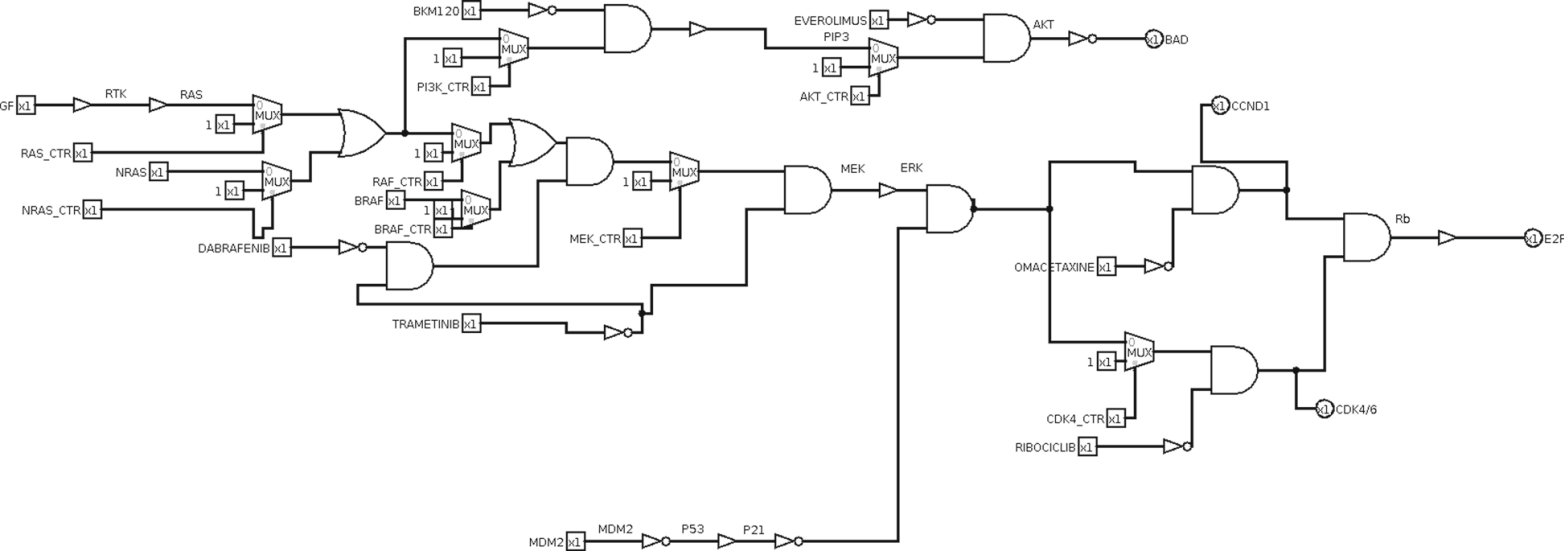
Melanoma pathway digital circuit with mutations and drugs. The circuit includes gene mutations modelled by multiplexers and the drugs modelled by a combination of NOT and AND gates, as discussed in the “Simulation” section

To identify the “pathogenic degree” of each mutation, we ran a simulation of the melanoma pathway logic circuit for each mutated gene. By applying mutation-target drugs (Fig. 16), simulation results highlight the best drug or drug combination for each mutation. By analysing simulation results, we identify the best drugs combination for the melanoma case study. We identified the set of drugs by selecting in the KEGG database suggested drugs that are known as “approved” in the DrugBank database. We take into account six drugs (Table 8): Trametinib targets MEK and BRAF, Dabrafenib targets RAF and BRAF, BKM12o targets PI3K gene, Omacetaxine targets CCND1 and Ribociclib targets CDK4/6. The drug vector is [Trametinib, Dabrafenib, BKM120, Everolimus, Omacetaxine, Ribociclib]. Given the fixed input test vector, simulations are carried out under each drug and each mutation to obtain the best drug combination for the selected disease. As for the NSCLC, combinations with no more than two drugs are considered to avoid significant side-effects. Table 9 shows results for drug discovery. Among selected drugs, Everolimus is the most effective being able to rectify almost all mutations. The best combination of two drugs is Everolimus/Ribociclib, achieving a cumulative score of S=-8 which is the maximum allowable value for the selected case study.

**Table 8:** Melanoma case study: Drugs, left column, and their corresponding targets, right column, related to the proposed case study

Drug	Target
Omacetaxine	CCND1
Ribociclib	CDK4/6
Dabrafenib	BRAF, RAF
Trametinib	BRAF,MEK
Everolimus	AKT
BKM120	PI3K

**Table 9:** Melanoma case study. Effects of drugs, in terms of the score defined in “Simulation” Section, on gene mutations

Drugs	BRAF	RAF	NRAS	RAS	AKT	PI3K	MEK	CDK4/6	Ranking
Score obtained by a single drug									
Everolimus	-1 → -1	-1 → -1	0 → -1	0 → -1	0 → -1	0 → -1	-1 → -1	0 → 0	-7
BKM120	-1 → -1	-1 → -1	0 → -1	0 → -1	0 → 0	0 → -1	-1 → -1	0 → 0	-6
Ribociclib	-1 → -1	-1 → -1	0 → 0	0 → 0	0 → 0	0 → 0	-1 → -1	0 → -1	-4
Trametinib	-1 → -1	-1 → -1	0 → 0	0 → 0	0 → 0	0 → 0	-1 → -1	0 → 0	-3
Dabrafenib	-1 → -1	-1 → -1	0 → 0	0 → 0	0 → 0	0 → 0	-1 → -1	0 → 0	-3
Omacetaxine	-1 → -1	-1 → -1	0 → 0	0 → 0	0 → 0	0 → 0	-1 → -1	0 → 0	-3
Score obtained by the combination of two drugs									
Everolimus/Ribociclib	-1 → -1	-1 → -1	0 → -1	0 → -1	0 → -1	0 → -1	-1 → -1	0 → -1	-8
Everolimus/BKM120	-1 → -1	-1 → -1	0 → -1	0 → -1	0 → -1	0 → -1	-1 → -1	0 → 0	-7

#### Melanoma miRNA therapeutics simulation results

**Fig. 17:**
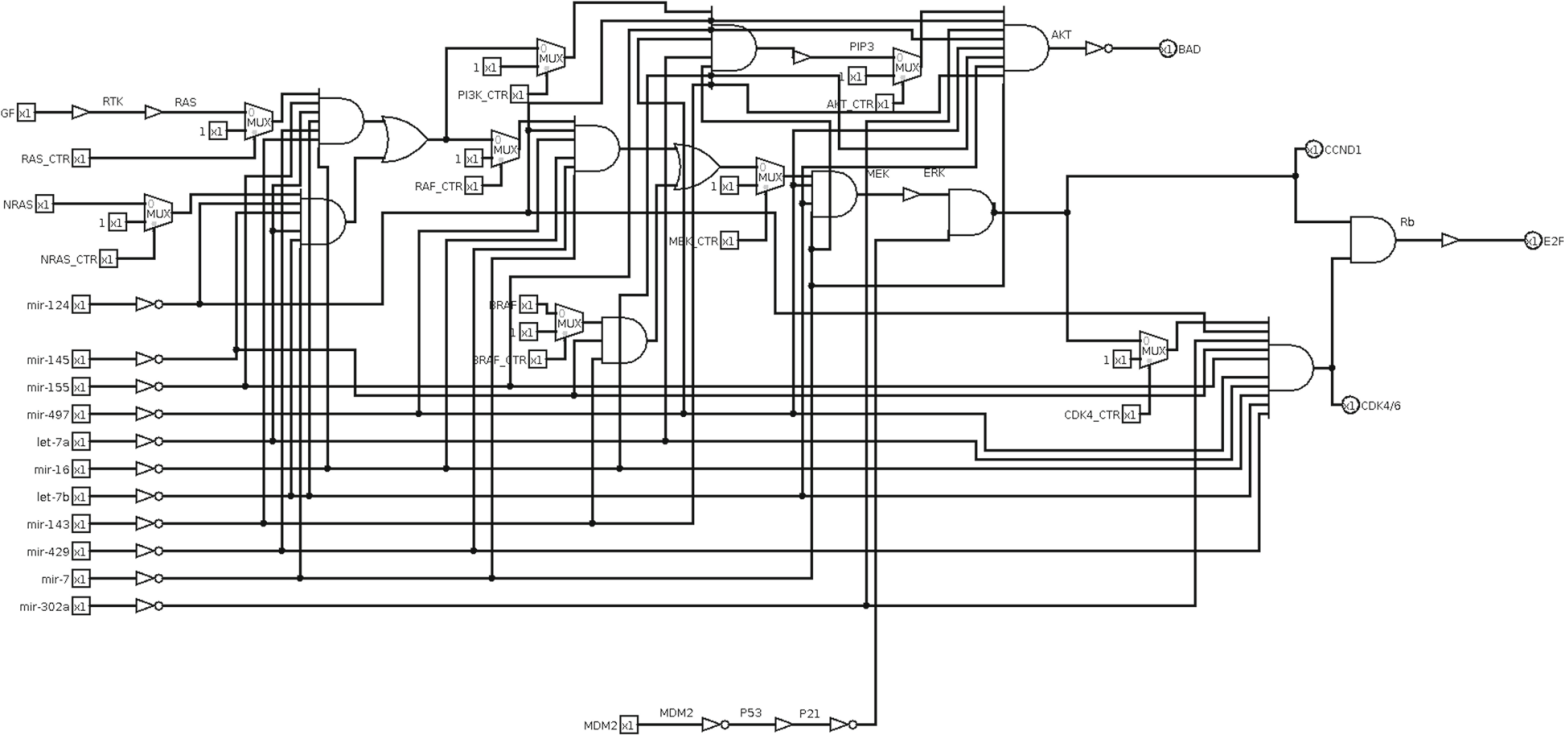
Melanoma pathway digital circuit with miRNA. Mutations are modelled by multiplexer and a combination of NOT and AND gates models the miRNA-target interactions

Also in melanoma disease, the role of miRNA is well-known [51]; for instance, authors investigated their potential use as nano molecules administered to cancer patients [52]. According to these studies, we tested miRNA therapeutics also in the melanoma case study. For miRNA therapeutics, we selected eleven miRNAs, filtering out results of miRNA-target validated interactions retrieved from miRTarBase. We obtained 637 miRNAs. In this case study, filtering results by miRTissue were not possible because the tissue filter for melanoma is not available. Among those interactions, we selected miRNAs addressing multiple targets. Some miRNAs share the same target, and therefore those miRNAs have the same effect on the pathway circuit. To avoid circuit redundancy, we selected a representative miRNA for each group. As a result of the post-filtering stage, we picked eleven miRNAs (Table 10). The analysis of the logic circuit with miRNA-target interactions (Fig. 17) reveals the best miRNA for each mutation. Table 11 lists results with miRNA therapeutics. It clearly shows several miRNAs give the best cumulative score of S=-8: let-7b, mir-155, mir-497, mir-16, mir-124, mir-302a. Recently, authors highlighted some of them for their role in cell cycle regulation, apoptosis, and cancer therapy [53]. In the following, we further discuss each of the miRNAs producing the best score. let-7b is often down-regulated in melanomas compared with healthy controls and it could also be involved in the transition from nevi to primary melanomas through the targeting of key cell cycle regulators [54]. An interesting study also shows that let-7 was down-regulated in vemurafenib (BRAF-V600 inhibitor) resistant melanoma cells. Introducing miR-7 in this cell, through miR-7 mimics, re-establishing their expression would reverse the drug resistance [53, 55]. Although miR-155 is described as

**Table 10:** Melanoma case study. miRNAs and their targets selected for the proposed case study

miRNA	Target
mir-124	CDK,AKT,NRAS,RAF,PI3K
mir-145	CDK,NRAS,BRAF
mir-155	CDK,KRAS,AKT,PI3K
mir-497	CDK,AKT,MEK,RAF,PI3K
let-7A	CDK,KRAS,NRAS,PI3K
mir-16	CDK,KRAS,AKT,RAF,PI3K
let-7b	CDK,AKT,NRAS,RAS,MEK
mir-143	KRAS,AKT,PI3K,BRAF
mir-429	CDK,KRAS,RAF
mir-7	KRAS,AKT,RAF,PI3K,MEK
mir-302a	CDK,AKT

**Table 11:** Melanoma case study. Effects of miRNAs, in terms of the score defined in “Simulation” Section, on gene mutations

miRNA	BRAF	RAF	NRAS	RAS	AKT	PI3K	MEK	CDK4/6	Ranking
Score obtained by a single miRNA									
mir-124	-1 → -1	-1 → -1	0 → -1	0 → -1	0 → -1	0 → -1	-1 → -1	0 → -1	-8
mir-155	-1 → -1	-1 → -1	0 → -1	0 → -1	0 → -1	0 → -1	-1 → -1	0 → -1	-8
mir-497	-1 → -1	-1 → -1	0 → -1	0 → -1	0 → -1	0 → -1	-1 → -1	0 → -1	-8
mir-16	-1 → -1	-1 → -1	0 → -1	0 → -1	0 → -1	0 → -1	-1 → -1	0 → -1	-8
let-7b	-1 → -1	-1 → -1	0 → -1	0 → -1	0 → -1	0 → -1	-1 → -1	0 → -1	-8
mir-302a	-1 → -1	-1 → -1	0 → -1	0 → -1	0 → -1	0 → -1	-1 → -1	0 → -1	-8
let-7a	-1 → -1	-1 → -1	0 → -1	0 → -1	0 → 0	0 → -1	-1 → -1	0 → -1	-7
mir-143	-1 → -1	-1 → -1	0 → 0	0 → -1	0 → -1	0 → -1	-1 → -1	0 → 0	-7
mir-7	-1 → -1	-1 → -1	0 → -1	0 → -1	0 → -1	0 → -1	-1 → -1	0 → 0	-7
mir-145	-1 → -1	-1 → -1	0 → -1	0 → 0	0 → 0	0 → 0	-1 → -1	0 → -1	-5
mir-429	-1 → -1	-1 → -1	0 → -1	0 → -1	0 → 0	0 → 0	-1 → -1	0 → -1	-5

an oncogenic miRNA in different cancer types, other studies support its role as pro-apoptotic in melanoma cells, suggesting a potential use as a therapeutic agent in decreasing tumor aggressiveness [56]. As regards mir-497-5p, literature provides a fascinating study of increased levels of this miRNA during treatment correlates with prolonged progression-free survival in 26 patients with metastatic cutaneous malignant melanoma treated with MAPK inhibitors [57]. Moreover, it has been shown to reduce cancer phenotype in the melanoma cell line (A375)[58]. Indeed a genome-scale lentiviral human miRNA expression library was used in the study and other miRNAs showed the same power to limit cancer cell proliferation, as miR-16, that also reported the best score in our method. Also, miR-124 and miR-302a are promising biomarkers in melanoma as their function is to inhibit proliferation and migration of cancer cell lines in melanoma [59, 60]. In particular, this second case study aims to offer a selection of miRNAs that are especially promising candidates for application in cancer therapy.

## Conclusions

In this paper a novel approach for in silico study of the effects of drugs and the development of miRNA therapeutics is proposed. The proposed method is based on a logic circuit representation of a cancer pathway and was tested on NSCLC case study. For this disease a suitable pathway was selected and then transformed into a logic circuit that allows the biologists and clinicians to simulate the effects of drugs and miRNAs on gene mutations. We found confirmation of the positive effects of drugs in contrasting cells non-apoptosis and proliferation, and we compared these effects with the effects of some miRNAs and miRNA families. We evaluated these effects by using a cumulative score based on the combination of these two mechanisms.

We can conclude that this pathway circuit method can help to guide in vitro and in vivo experiments towards the most effective therapeutic miRNAs/drugs in human diseases, starting from the corresponding pathway. We are actually investigating a method to automatically transform the signalling pathway to the corresponding digital circuit.

## Abbreviations

ALK: Anaplastyc Lymphoma Kinase
ASO: AntiSense Oligonucleotides
ECrel: Enzyme-Enzyme relation
GErel: Gene Expression relation
KEGG: Kyoto Encyclopedia of Genes and Genomes
KGML: KEGG Markup Language
LNA: Locked Nucleic Acid
miRNA: microRNA
mRNA: messenger RNA
NSCLC: Non-Small Cell Lung Cancer
PCrel: Protein-Compound relation
PPrel: Protein-Protein relation
RNA: RiboNucleic Acid
RNAi: RNA interference.
